# Integrative mRNA and miRNA Expression Profiles from Developing Zebrafish Head Highlight Brain-Preference Genes and Regulatory Networks

**DOI:** 10.1007/s12035-024-04364-5

**Published:** 2024-07-31

**Authors:** Shuqiang Zhang, Jian Yang, Jie Xu, Jing Li, Lian Xu, Nana Jin, Xiaoyu Li

**Affiliations:** 1 College of Life Science, Henan Normal University, Xinxiang, 453007 Henan China; 2 Key Laboratory of Neuroregeneration of Jiangsu and Ministry of Education, Co-Innovation Center of Neuroregeneration, NMPA Key Laboratory for Researchand, Evaluation of Tissue Engineering Technology Products , Nantong University, Nantong, 226001 China; 3 Institute for Translational Neuroscience, the Second Affiliated Hospital of Nantong University, Nantong University, Nantong, 226001 Jiangsu China; 4 The School of Medical Humanities, Xinxiang Medical University, Xinxiang, 453003 Henan China

**Keywords:** Head development, Zebrafish, Transcriptome, miRNA, mRNA

## Abstract

Zebrafish is an emerging animal model for studying molecular mechanism underlying neurodevelopmental disorder due to its advantage characters. miRNAs are small non-coding RNAs that play a key role in brain development. Understanding of dynamic transcriptional and post-transcriptional molecules and their regulation during the head development is important for the study of neurodevelopmental disorder. In this study, we performed the high-throughput sequencing of mRNAs and miRNAs in developing zebrafish head from pharyngula to early larval stages and carried out bioinformatic analysis including differential expression and functional enrichment as well as joint analysis of miRNAs and mRNAs, and also compared with other related public sequencing datasets to aid our interpretation. A large number of differential expression genes with a large fold change were detected during the head development. Further clustering and functional enrichment analyses indicated that genes in late stage were most related with synaptic signaling. Overlap test analysis showed a significant enrichment of brain-preference and synapse-associated gene set in the head transcriptome compared with the whole embryo transcriptome. We also constructed miRNA-mRNA network for those brain-preference genes and focused on those densely connected network components. CRISPR-Cas9-mediated *snap25b* mutants led to embryonic development defects and decreases locomotor activity. Altogether, the present study provides developmental profiles of head-enriched mRNAs and miRNAs at three critical windows for nervous system development, which may contribute to the study of neurodevelopmental disorder.

**Supplementary Information:**

The online version contains supplementary material available at 10.1007/s12035-024-04364-5.

## Introduction

Zebrafish (*Danio rerio*) have remarkable advantages such as high genomic conservation with human genes, high fecundity, ex vivo embryogenesis, quick development, and embryonic translucency [1]. The zebrafish embryonic stages are divided into seven broad periods, including pharyngula (24–48 h post-fertilization, hours (h) in this study refers to the post-fertilization) and hatching periods (48–72 h) and most developmental processes occur during the first 3 days [2]. Unlike limited regenerative capacity in mammals, lower vertebrates, such as zebrafish, could robustly regenerate a range of organ after injury, including spinal cord, heart, fin, and retina [3]. These advantages have made the zebrafish as an ideal animal model for the study of numerous areas, such as vertebrate development, aging, regenerative medicine, and human diseases (neurologic and neurodevelopmental disorders) [1, 3, 4]. Thus, understanding the molecular and cellular dynamics during the zebrafish development, specifically developing nervous system, is critical.

With the advancement of the high-throughput sequencing technologies, pioneer studies have investigated multiple aspects of the zebrafish development, including epigenomic level, transcription level, protein level using multi-omics at bulk and/or single-cell resolution, providing critical biological insights [1, 5–8]. However, most of studies used the whole embryos or adult tissues (e.g., brain and eye) and a special focus on the development of the nervous system remains limited. MicroRNAs (miRNAs) are small non-coding RNAs with the size of about 22 nucleotides (nt), which post-transcriptionally regulate gene expression and involve a range of cellular processes such as organismal development and pathology [9]. Most miRNAs are conserved in animals, but present a highly tissue-specific manner. For example, miR-9 is highly expressed in both the developing and adult vertebrate brain [10]. Previous studies have explored miRNAs from different tissues in adult zebrafish or pooled whole embryo using next-generation technology [11].

The eye and brain are the relatively large organs in the zebrafish head [12]. Thus, we selected the time points based on the eye development. The early pharyngula stage (24 h) is the key time point for diverse vertebrate embryonic morphology, including the nervous system that is hollow and expanded anteriorly [2]. For photoreceptor morphogenesis, the photoreceptor cell layer can be distinguished by 48 h but the differentiation of morphologically distinct photoreceptor types becomes apparent by 96 h [2]. In this study, we applied both mRNA and miRNA sequencing of developing head of zebrafish spanning three key time points (24, 48, and 96 h) to decode dynamic expression of miRNAs and mRNAs. Comparison with other public tissue sequencing datasets highlighted dynamic nervous system preferred expression genes and miRNAs, and integrated with miRNA and mRNA profiles unveiled potential miRNA-mRNA regulatory networks. To verify the function of brain specific gene, we employed CRISPR-Cas9 technology to construct *snap25b* mutants and study the potential function of *snap25b* during zebrafish development. This study not only provides a valuable resource for the nervous system development but also shed insights into molecular dynamic and miRNA-mRNA regulation for the study of head development as well as neurodevelopmental disorders.

## Materials and Methods

### Animals

Wild-type AB zebrafish (China Zebrafish Resource Center, Wuhan, China), aged 6–12 months, were used to breed embryos for this study. The zebrafish were cultured in a recirculating aquaculture system (ESEN, Beijing, China) under the following conditions: PH level of 7–8, electrical conductivity of 500–550 μS/cm, a 14/10 h light/dark photoperiod, and a constant temperature of 28.5 ℃. Embryos after fertilization were cultured in E3 medium, which was replaced daily. Larvae aged 6–15 days were kept in small tanks, fed twice daily with paramecia, and had their water changed daily. After 15 days, juveniles were transferred to the breeding system and fed a small amount of live brine shrimp immediately after hatching. Adult zebrafish were fed live brine shrimp twice daily, with an amount that could be consumed completely within 15 min. The heads of the embryos at development stages (24 h, 48 h, and 96 h) were prepared in a cultured dish with cold E3 medium under a stereomicroscope and immediately frozen in liquid nitrogen. All animal experiments were approved by the Animal Care and Use Committee of Nantong University (approval ID: S20220313-001).

### Tissue Preparation

To isolate early embryonic head tissue, we first manually removed chorions using forceps and then placed in a cell culture dish with a diameter of 12 mm and added appropriate amount of E3 medium. After sufficient head tissue samples were collected, the tissues were transferred to a new EP tube. After removing culture fluid, the tissues were quickly flash frozen into liquid nitrogen and stored at − 80 ℃.

### RNA-seq Library Preparation and Sequencing

Total RNA was extracted from head tissues at the studied embryonic developmental stages (24 h, 48 h, and 96 h) using TRIzol reagent according to the manufacturer’s protocol. rRNA-depleted library construction and sequencing were performed by the Gene Denovo Biotechnology Co. (Guangzhou, China). Simply, ribosomal RNA was firstly removed using Ribo-ZeroTM Magnetic Kit (Epicentre) and then the remaining RNA was randomly fragmented into short fragments (200–500 nt). The fragmented RNA was used as a template for synthesizing the first strand of cDNA using random primers. Subsequently, a second cDNA strand was synthesized by adding buffer, RNase H, DNA polymerase I, and dNTPs with dTTP were replaced by dUTP. The cDNA fragments were then purified, end repaired, poly(A) added, and sequencing adapter ligation. The second strand was then degraded using uracil-N-glycosylase (UNG) enzyme. Fragment size selection was performed using agarose gel electrophoresis, followed by PCR amplification. High-through sequencing was performed on the Illumina HiSeq 4000.

### miRNA-seq Library Preparation and Sequencing

For miRNA sequencing, library preparation and sequencing were performed by Gene Denovo Biotechnology CO. (Guangzhou, China). Simply, the RNA fragments within the size of 18–30 nt were enriched by polyacrylamide gel electrophoresis (PAGE). Then 3′ and 5′ adapters were ligated separately, followed by reverse transcription and PCR amplification for library construction. High-through sequencing was performed on the Illumina HiSeq 4000.

### RNA-seq and miRNA-seq Quantification

For RNA-seq, fastp was used to trim adapter and filter low-quality reads [13]. Then, clean reads were mapped against to the reference (GRCz10 Ensembl release 91) using Tophat2 (v2.1.1) [14]. RSEM software was used to estimate gene abundance [15]. For miRNA-seq, miRNAs in zebrafish genome were retrieved from miRbase database (https://www.mirbase.org/). miRge3.0 pipeline [16] was employed to analyze miRNA expression profiles, including sequence alignment and quantification.

### Differential Expression Analysis

To perform differential expression analysis of two developmental stages, DESeq2 R package [17] was used. For mRNA, genes with adjusted *P*-value < 0.001 and |log2-transformed fold-change|≥ 2 were defined as a differential expression gene (DEG). For miRNA, those miRNAs with adjusted *P*-value < 0.05 and |log2-transformed fold-change|≥ 1 were defined as differentially expressed miRNAs. Clustering and visualization of DEGs or differentially expressed miRNAs were conducted using the ComplexHeatmap Package [18].

### Tissue or Stage-Specific Gene Screening

Tau (*τ*) metric implemented in Tspex [19] was used to calculate tissue or stage-specific index. For a gene with tau ≥ 0.8 and normalized FPKM or TPM greater than 1 was defined as a specific expression gene at a tissue or a stage.

### miRNA Target Analysis

High abundance of miRNAs (maximum RPM > 100) and mRNAs (maximum FPKM > 10) were selected. mRNAs with differential expression by pairwise comparison between two developmental stages and high preference (tau > 0.7) of expression in adult brain were further focused. miRNA-mRNA interacting pairs were retrieved from the miRWalk 2.0 database [20] (http://mirwalk.umm.uni-heidelberg.de/) which predicts miRNA targets using a machine learning algorithm containing experimentally verified miRNA-target interactions. Then, we calculated Pearson’s correlation based on the expression between miRNA and mRNA target. miRNA-mRNA interacting pairs with a significant negative correlation (*r* <  − 0.4 and *p* value < 0.05) were kept. Next, final mRNA targets were uploaded to the online annotation and enrichment analysis tool, Metascape (https://metascape.org/gp/index.html) [21], for gene-set enrichment and protein–protein interaction (PPI) analyses. Densely connected network components (DCNC) of PPI network were determined by the Metascape. Interaction of genes in the DCNCs and their potential miRNAs was visualized using NGenomeSyn [22]. The network of highly connected miRNAs and mRNAs was visualized using the Cytoscape [23].

### Public Data Reanalysis

To enhance our knowledge about molecular signature of developing head in zebrafish, we also reanalyzed and compared with other public datasets including time-course RNA-seq datasets of developmental stages from pooled embryos and/or larvae as well as adult tissues, and miRNA-seq dataset of adult tissues [7, 8, 11, 24]. For public RNA-seq, expression profiles were directly retrieved from GEO database under accessions, GSE134055 and GSE171906. For public miRNA-seq dataset (GSE57169), we used the same reference and quantification pipeline from raw FASTQ files as we mentioned above.

### RT-qPCR Validation

Reverse transcription quantitative PCR (RT-qPCR) was employed to validate mRNA and miRNA expression. For RT-qPCR validation of mRNAs, we employed the standard procedure. Simply, the total RNA was extracted from the head using the RNA isolater Total RNA Extraction Reagent (R401, Vazyme, China) and then reverse transcribed into cDNA using HiScript II 1st Strand cDNA Synthesis Kit (+ gDNA wiper, R212, Vazyme, China). For RT-qPCR validation of miRNAs, the total RNA was extracted and then reverse transcribed into cDNA using mRNA 1st Strand cDNA Synthesis Kit (by tailing A). Real-time qPCR was then performed using Taq Pro Universal SYBR qPCR Master Mix Kit (Q712, Vazyme, China) according to the manual protocol. Relative expression was calculated using the method of 2^−∆∆Ct^. The primers for the selected mRNAs and miRNAs are listed in Table S6.

### *Whole-Mount *in situ* Hybridization*

To confirm the tissue distribution of selected genes, we performed whole-mount in situ hybridization (WISH) of zebrafish embryos according to the procedure described in the study by Wang et al. [25]. Simply, the specific probe primers were designed using the Primer3web (v4.1.0, https://primer3.ut.ee) and synthesized. We then prepared digoxygenin-labeled target gene antisense mRNA probes through transcription in vitro. Subsequently, embryos collected at 96 h were hybridized with target gene probes by several major steps including fixation at 4% solution of paraformaldehyde (PFA) and then digestion using the proteinase K and incubation with a pre-hybridized mix. Lastly, the alkaline phosphatase (AP)-conjugated antibody against digoxigenin (anti-Digoxigenin-AP, 11,093,274,910, Roche) and the AP-substrate NBT/BCIP solution (11,681,451,001, Roche) were employed to detect the target gene expression. The primers used for probe synthesis are listed below:

dpysl5b-F: CATTGCTCGAATCCACGCAG; dpysl5b-R: TCTCTCCCAGATGACGCTCA.

aldocb-F: GGCTGGAACAAACGGAGAGA; aldocb-R: TTCAGGTTGACAGACGCCTC.

snap25b-F: GCATTCGGTGACCCAAATCA; snap25b-R: GGACACGGACATAGACCACA.

### Zebrafish snap25b Mutant Preparation

To generate *snap25b* mutant zebrafish, we employed CRISPR-Cas9 technology according to the protocol developed by Welker et al. [26]. Briefly, we designed a single-guide RNA (sgRNA) using the online website (https://www.crisprscan.org/) and further synthesized in vitro as follows. First, the sgDNA was cloned by PCR with a forward primer containing the *snap25b* target site (5′- AAGGTGGTGGTCAGTCCTGT -3′) and a universal reverse primer. Subsequently, about 5 μL mixture of 1 μL Cas9 protein (E365-01A, novoprotein, China) and 100 ng/L *snap25b* sgRNA was microinjected into zebrafish embryos at 1-cell stage. The *snap25b* mutation in F0 embryos was examined via PCR using designed sequencing primers (forward primer: CCAAGGGTGCCAGTATTAGTG; reverse primer: AGCGAGCTATAGTGTTGTGC) and the amplified products containing the targeting site were sequenced. After confirming the *snap25b* mutation, F0 embryos were bred into adult zebrafish and then outcrossed with the AB wild-type and their embryos (termed F1) were subjected to genotyping. To obtain reliable *snap25b* mutants, the F1 adult zebrafish were further outcrossed with the AB wild-type and their embryos were termed F2. F2 larvae were then used for observation of viability and morphology (96 hpf), and swimming behavior test (8 dpf).

## Results

### Dynamic Expression of mRNAs in the Developing Head of Zebrafish

**Fig. 1:**
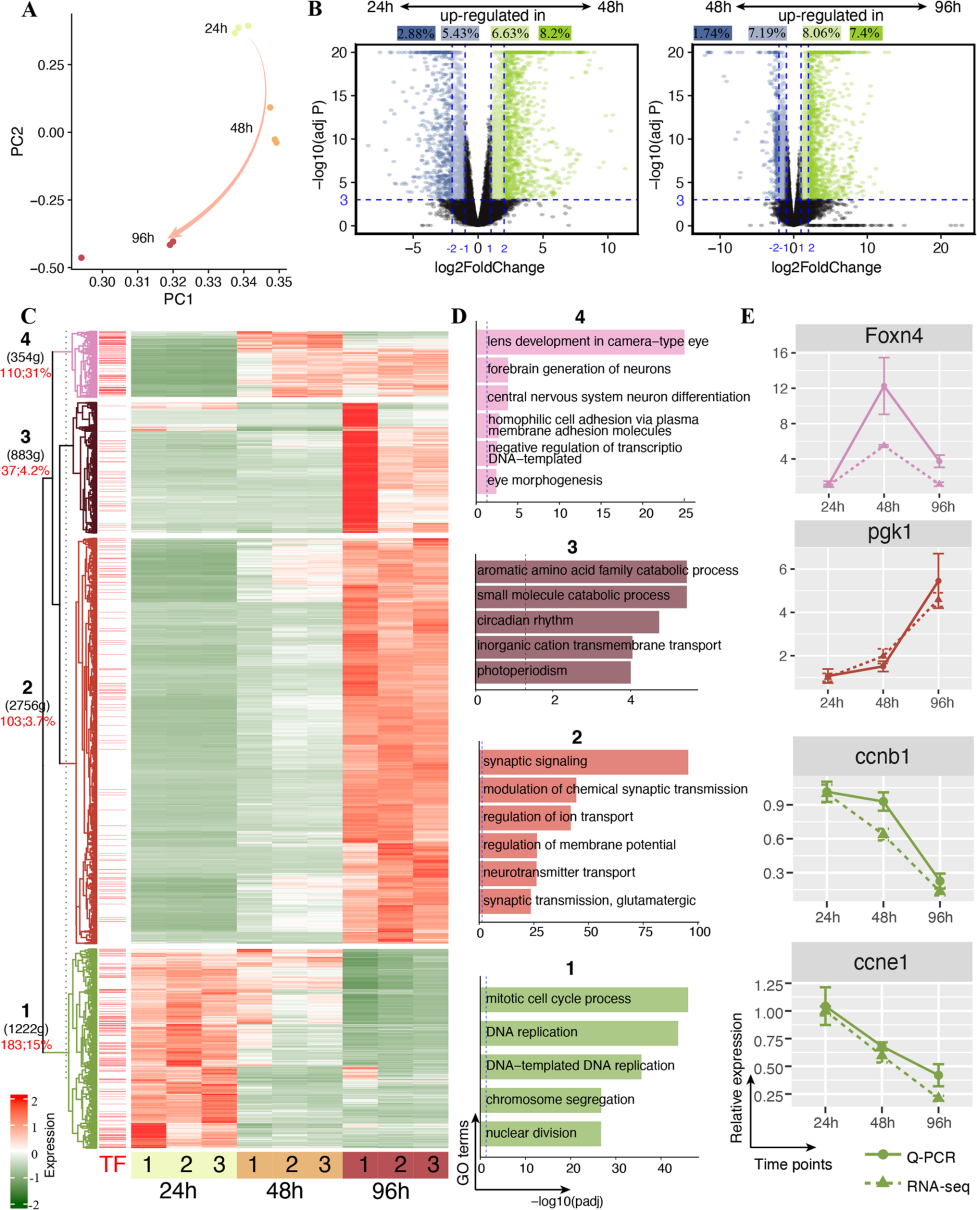
Differentially expressed protein-coding genes (DEGs) in developing zebrafish head transcriptome. **A** Sample distribution according to the PCA analysis. **B** Volcano plots of DEGs by neighbor time point comparisons. Dark colors depict large change of genes (LFC ≥ 2) while light colors depict moderate change of genes (1 ≤ LFC < 2). **C** Expression patterns of DEGs by pairwise comparisons and were divided into four clusters. **D** GO biological process enrichment analysis of DEGs in four clusters. **E** RT-qPCR validation of four genes in RNA-seq datasets

To study dynamic expression of genes related with nervous system development, we carried out a high-coverage RNA sequencing of zebrafish head at three critical windows of developmental process, 24-h post-fertilization (h), 48 h, and 96 h using a stranded-specific manner, yielding 117–187 million reads per sample. A total of 15,728–19,680 protein-coding genes per sample were detected (a gene with FPKM ≥ 1 was defined as expressed). Principal component analysis (PCA) showed three separate distinct clusters with three biological replicates in each time point clustered together, indicating obvious distinct molecular signatures among 24 h, 48 h, and 96 h (Fig. 1A). Next, we performed pairwise differential expression analysis to investigate dynamic expression of genes during the head development. We found a large number of differential expression genes (DEGs) by neighboring comparisons (48 h vs. 24 h and 96 h vs. 48 h, Fig. 1B) and with more than 20% of the expressed genes were detected under a relax threshold (4521 DEGs in 48 h vs. 24 h, 5067 DEGs in 96 h vs. 48 h; adjusted *P*-value < 0.001 and |log-transformed fold-change|≥ 1). Notably, we found that nearly 50% of up-regulated genes had a large fold change (≥ 4) while most down-regulated genes had a small fold-change (< 4, Fig. 1B). We then clustered 5648 DEGs with a large fold-change (≥ 4) in comparisons of “48 h vs. 24 h” and/or “96 h vs. 48 h” and divided into four clusters based on the expression pattern using the ComplexHeatmap package (Fig. 1C). Most DEGs (2756 out of 5648; 48.8%) presented in cluster 2 with the expression dramatically increased at 96 h and significantly enriched in terms related with neuronal development, including “synaptic signaling” and “neurotransmitter transport” (Fig. 1D). Genes (*n* = 1222) in cluster 1 showed decreased expression along with the development and significantly enriched in terms related with cell cycle (GO:0007049; 156 genes), including *mcm6*, *cdk1*, *cdk2*, *ccnd1*, *ccnb1*, and *ccne1* (Fig. 1C and 1D and Table S1). RT-qPCR validation of seven cell-cycle-related genes (*mcm6*, *cdk1*, *cdk2*, *ccna2*, *ccnb1*, *ccnd1*, and *ccne1*) showed a consistent expression pattern between RNA-seq data and experiment results (Figs. 1E and S1). Genes (*n* = 354) in cluster 4 showed increased and peaked expression at 48 h and significantly enriched in terms of eye development, including “lens development in camera-type eye” and “eye morphogenesis,” such as genes encoding crystalline proteins (*crybb1l2*, *cryba4*, *cryba4*, *cryba4*, and *cryba4*) (Fig. 1D). Notably, we found the largest percent (110 out of 354; 31%) of transcription factors (TFs) in cluster 4 than other three clusters (3.7%–15%, Fig. 1C). Of these TFs in cluster 4, 10 TFs showed a high expression in cell population related with retinal differentiation and/or photoreceptors based on the single-cell transcriptome for zebrafish development [5] (also presented in Fig. S2), including *foxn4*, *neurod1*, *crx*, *atoh7*, and *hes2.2* (Fig. 1C). In general, up-regulated genes are primarily associated with brain and eye development while down-regulated genes are predominantly involved in the cell cycle during the head development.

### Significant Enrichment of Developmental Brain-Preference Genes

**Fig. 2:**
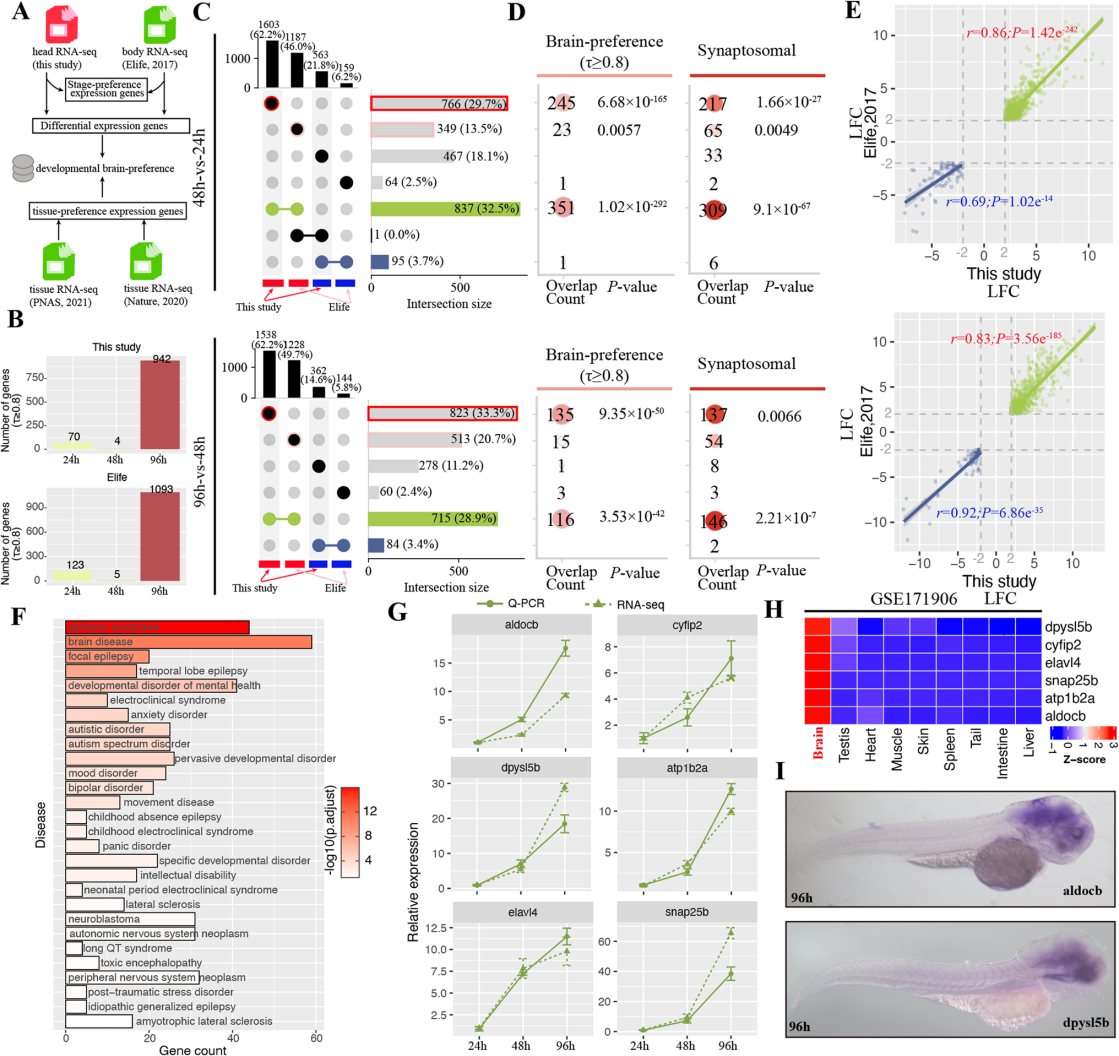
Significant enrichment of brain-preference genes in head transcriptome compared to that in the whole embryo RNA-seq. **A** Overview of identification of developmental brain-preference genes by integrating datasets from this study and other publication. **B** Number of stage-specific expression genes. **C** The upset plots of up- and down-regulated DEGs between head and whole embryo developmental RNA-seq. **D** Significant enrichment of brain-preference and synaptosome genes in DEGs only detected from head transcriptome. **E** High correlation of DEGs detected both in head and whole embryo transcriptome based on log2-transformed fold-change. **F** Disease enrichment of developmental brain-preference genes. **G** RT-qPCR validation of genes with preferred expression in brain. **H** In situ hybridization of protein-coding genes, *aldocb* and *dpysl5b*

To confirm our data accuracy and assess whether sequencing of zebrafish head tissue could well represent more developmental brain-specific genes, we next compared with other published datasets, a pioneer study of time-series expression profile of whole embryo [7] and two studies of tissue expression in adult zebrafish [8, 24] (Fig. 2A). The same time points (24 h, 48 h, and 96 h) of developmental expression profile were analyzed. Tau index is usually used for measurement of degree of a gene with expression preferred in one or several tissues restricted based on the expression profile [19]. We firstly calculated tau index for screening genes with stage-specific expression. We found a similar trend of number of genes with stage-specific expression between dataset from our study and White et al. study [7] and only several genes (< 10) presenting specifically expressed at 48 h whereas numerous genes (> 900) presenting specifically expressed at 96 h (tau ≥ 0.8, Fig. 2B). We next performed differential expression analysis using the same threshold for White et al. study and compared with the result of our study. Relative more DEGs were detected in our study and the number of shared up-regulated genes was more than the number of shared down-regulated genes (Fig. 2C). Comparison of log-transformed fold-change between these two developmental datasets showed a significant positive correlation, specifically shared up-regulated genes (*r* = 0.69–0.92; *P* < 1e^−10^; Fig. 2E). Moreover, we found that a large number of DEGs was only detected in head transcriptome than that only detected in the whole embryos using the same threshold (e.g., 766 up-regulated genes in the former while 349 up-regulated genes in the latter in comparison of 48 h and 24 h, Fig. 2C). Two public RNA-seq dataset [8, 24] (GEO accessions: GSE134055 and GSE171906) sequencing distinct tissues in adult zebrafish were used to determine that whether head-preference DEGs were high expression in brain. A total of 990 genes with tau index greater than 0.8 in brain tissue commonly detected in these two datasets were screened as a brain-preference gene set, including 64 TFs (e.g., *olig2*, *neurod1*, *foxg1a*, *nkx2.2a*, *otx2*, and *myt1la*) (Table S2). We then performed over-representation analysis (ORA) for genes in categories corresponding to the right bar plots in Fig. 2C using the hypergeometric test. The result showed both significant overlap of head-preference and whole embryo-preference up-regulated genes with brain-preference genes (*p* < 0.05), but a larger number (245 genes in head-preference versus 23 genes in whole body in comparison of 48 h vs. 24 h; 135 genes in head-preference versus 15 in whole body in comparison of 96 h vs. 48 h) of head-preference up-regulated genes were presented in the brain-preference gene-set (Fig. 2D). We also collected 3640 synapse proteins in zebrafish [27] and also performed ORA analysis. Consistent with the result of brain-preference gene set, a larger number of overlapped genes between head-preference and synapse-related genes compared to that in whole embryo preference (Fig. 2D). Gene Ontology (GO) enrichment analysis of genes in comparison of 48 h and 24 h showed head-preference up-regulated genes mostly enriched in terms of “synaptic signaling” while whole embryos-preference genes mostly enriched in terms of “muscle structure development” or “cellular amine metabolic process” (Fig. S3). Together, these pieces of evidences supported our data accuracy and indicated a higher detection of brain-preference genes in developing head transcriptome than that in whole embryos.

To show the importance of head-enriched genes in brain development and their correlation with human brain disease, we performed disease enrichment analysis of 5648 DEGs detected in developing head transcriptome against disease database (DOSE) using the ClusterProfiler package [28]. There were 771 out of 5648 DEGs that were successfully converted into human orthologs and significantly enriched in neuropsychiatric disorders (e.g., epilepsy and anxiety), including synaptic vesicle glycoprotein 2A (*sv2a*) and synapse proteins (e.g., *syt1*) (Fig. 2F and Table S3). Six genes (*aldocb*, *cyfip2*, *dpysl5b*, *atp1b2a*, *elavl4*, and *snap25b*) were chosen for RT-qPCR validation and showed a high consistence with RNA-seq data (Fig. 2G). Tissue expression profile also showed a high expression of these six genes in brain than other tissues (Fig. 2H). In addition, RNA in situ hybridization experiment of two genes, *aldocb* and *dpysl5b*, also confirmed a high expression in embryonic brain (Fig. 2I). Collectively, our data and integrated with other adult tissue expression profiles highlight numerous developmental brain-preference genes.

### CRISPR-Cas9-Based snap25b Knockout Causes Embryonic Development Defects and Decreases Locomotor Activity

**Fig. 3:**
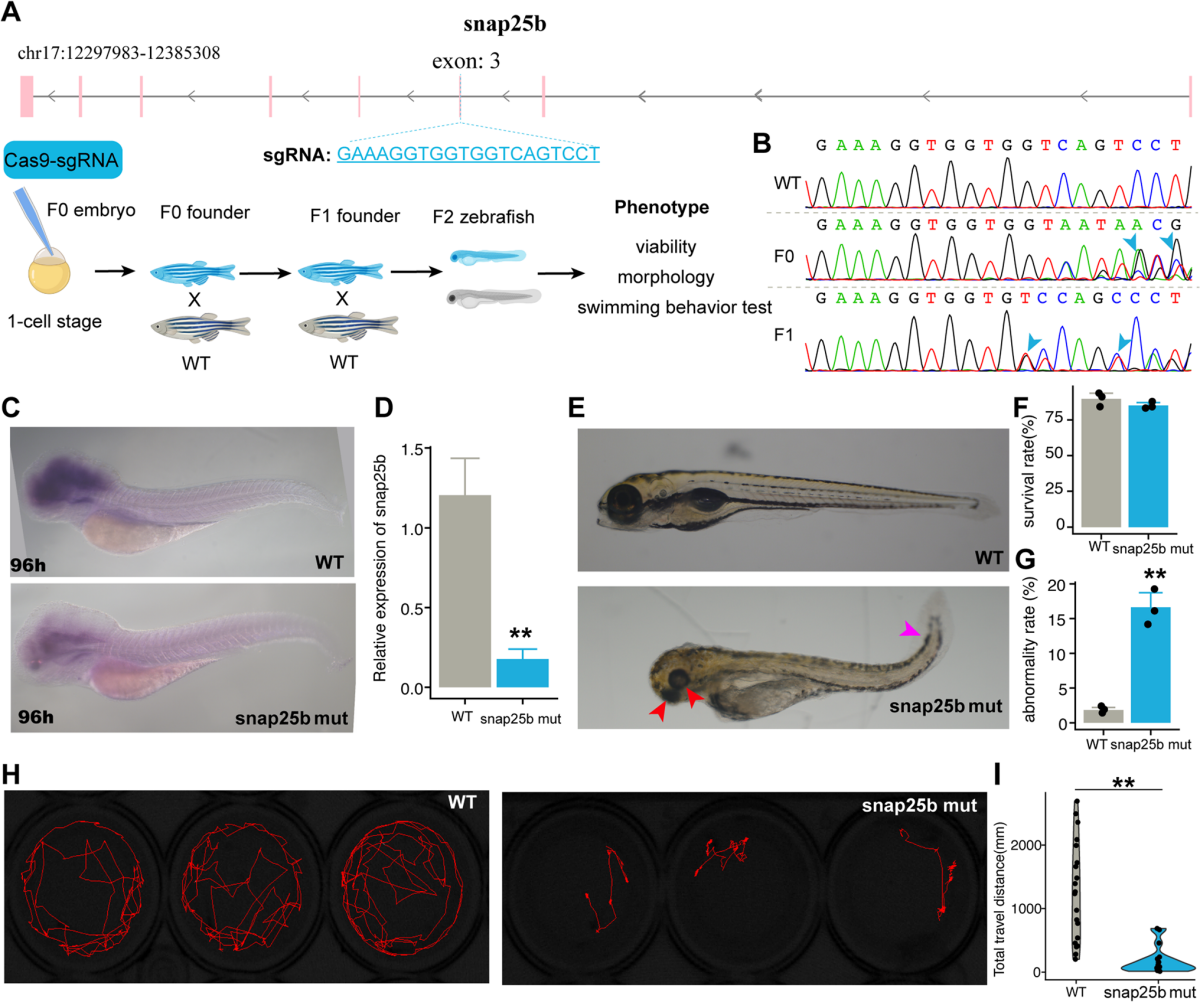
*Snap25b *knockout causes embryonic development defects and decreases swimming activity. **A **The *snap25b* gene structure and the schema for CRISPR-Cas9 technology to reduce *snap25b* expression in zebrafish. **B** Sanger sequencing confirms the mutations caused by CRIPSR-Cas9 in F0 and F1 generations. Primary sequence was shown. **C** In situ hybridization of *snap25b* in wild-type (WT) and *snap25b* mutant zebrafish. **D** The expression of *snap25b* by RT-qPCR in the head of wild-type and *snap25b* mutant zebrafish. **E** The representative abnormal phenotype included developmental delay, curled tail, and agenesis of eye. **F** The summary of survival rate in wild-type and *snap25b* mutants. **G** The summary of abnormality rate in wild-type and *snap25b* mutants. The Danio Vision system recorded the swimming behavior of wild-type and snap25b mutants (**H**) and analysis of total swimming distance (**I**). Data are presented as mean ± SD, *n* = 3 or *n* = 15–18; ** *p* < 0.01

In validation of gene expression by RT-qPCR, we noticed that *snap25b* showed the most increase of expression during the head development in zebrafish (Fig. 2G). *Snap25b*, an ortholog of mammalian *Snap25*, is a component of SNARE (soluble N-ethylmaleimide sensitive factor attachment protein receptor) complex and involves in the process of neurotransmitter release [29]. It has been shown that *Snap25* is a causative factor for developmental and epileptic encephalopathies of infancy and childhood [29]. To explore the function of *snap25b* in embryonic development, we employed the CRISPR-Cas9 technology to construct *snap25b* mutants (Fig. 3A). We designed a single-guide RNA fragment (sgRNA, targeting the exon 3 of *snap25b*), synthesized Cas9/sgRNA ribonucleoprotein, injected into 1-cell stage embryo (termed F0 generation) and further outcrossed twice with wild-type zebrafish to generate reliable *snap25b* mutants (termed F1 and F2, Fig. 3A). We tested the effectiveness of the *snap25b* knockout by the Sanger sequencing. The result showed the multiple peak curves appeared behind the target site in *snap25b* mutant (F1) compared with the wild-type (Figs. 3B and S4), indicating the successful mutation targeting exon 3. The wild-type *snap25b* consists 612 bp and could translate into a protein with 203 a.a. We found that *snap25b* mutants in F1 zebrafish had a deletion of 2 bp in the coding region, leading to a truncated protein with length of 102 a.a. The ISH and RT-qPCR experiments showed the expression of *snap25b* in the head of *snap25b* mutants were obviously decreased compared with the wild type at 96 hpf (Fig. 3C and 3D), indicating a successful construct of *snap25b* mutants. We next investigated larvae viability and embryonic morphology change in *snap25b* mutants at 96 hpf. We found a decrease of viability in *snap25b* mutants (85.1%) but without a significant difference compared with the wild type (89.7%, Fig. 3F). However, compared with the wild type (1.8%), a significant increase of malformation rate in *snap25b* mutants (16.6%) was observed (Fig. 3C). The abnormal morphology included developmental delay, curled tail, and agenesis of eye (Fig. 3E and 3G). Next, we assessed the locomotor activity in *snap25b* mutants at 8 dpf using the Danio Vision system. We found a significant decrease of total swimming distance in *snap25b* mutants compared with the wild type (Fig. 3H and 3I), indicating locomotor dysfunction. Taken together, the above data indicate that *snap25b* knockout led to embryonic development defects and decreased locomotor activity.

### Dynamic Expression of MiRNAs During Head Development

**Fig. 4:**
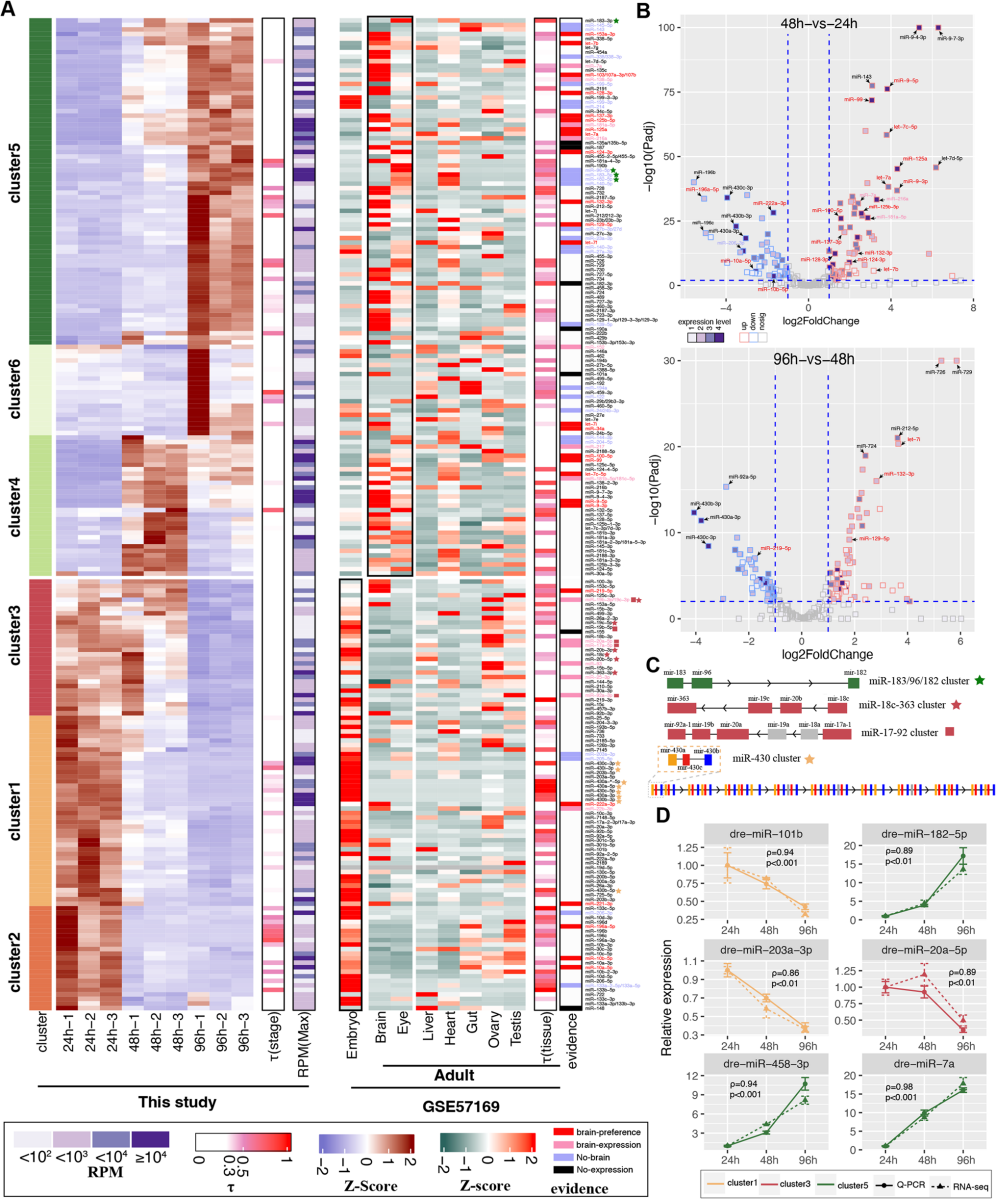
Development-related miRNAs in zebrafish brain. **A** Expression patterns of differentially expressed miRNAs by pairwise comparisons, expression of miRNAs in adult tissues, as well as the evidence supported by the RNA in situ hybridization experiment. **B** Volcano plots depict differentially expressed miRNAs in comparison of 48 h vs. 24 h and 96 h vs. 48 h. **C** Illustration locus of four miRNA clusters. Colors in upper three clusters indicated the expression patterns in Fig. 3a, and gray color indicated miRNAs without differential expression. **D** RT-qPCR validation of six miRNAs in RNA-seq datasets

We also conducted miRNA sequencing (miRNA-Seq) for the same counterpart to explore miRNA-mRNA networks related with eye or brain development. A public miRNA-Seq dataset of distinct adult tissues with brain and eye, in adult zebrafish as well as pooled embryos [11], were reanalyzed and Mireg3.0 pipeline was employed to analyze the miRNA-seq datasets, including trimming and quantification [16]. A total of 306 unique known mature miRNAs were detected in developing head transcriptome with a relatively high abundance (maximum read count ≥ 50). miRNAs with most abundance ranked the top five at 24 h were miR-430a/b-3p, miR-30e-5p, miR-206-3p, and miR-199-5p, while miR-181a-5p, miR-199-5p, miR-9-5p/3p, miR-183-5p, and miR-125b-5p ranked the top five at 48 h and/or 96 h. miR-206 was also shown most abundant in the early stage (postnatal 1 day, P1d) among these genes during rat retina development [30]. In total, 218 miRNAs showed a significantly differential expression (|log_2_^Fold−Change^|≥ 1 and adjusted *P*-value < 0.05) during the head development by pairwise comparison and was divided into six clusters based on their expression patterns (named clusters 1–6, Fig. 4A, Table S4). miRNAs in clusters 1, 2, and 3 showed higher expression at 24 h, which decreased as development progressed. In contrast, miRNAs in clusters 4, 5, and 6 exhibited higher expression at 48 h and/or 96 h (Fig. 4A). Unlike a large number of mRNAs with a stage-specific expression at 96 h, a smaller tau index was observed and most miRNAs with a large tau index were relatively low expressed (RPM < 1000, Figs. 1C and 4A), indicating most miRNAs were broadly expressed from 24 to 96 h. Another miRNA-Seq dataset showed that most miRNAs in clusters 1, 2, and 3 were also higher expression in embryos than that in adult tissues (e.g., brain and eye), and most miRNAs in clusters 4, 5, and 6 showed a brain and/or eye preference, such as miR-9, let-7a, and miR-125a (Fig. 4A). RT-qPCR validation of expression for six miRNAs (miR-101b, miR-182-5p, miR-203a-3p, miR-20a-5p, miR-458-3p, and miR-7a) during head development showed a high consistence of RT-qPCR result with sequencing data (*r* ≥ 0.86; *p* < 0.01, Fig. 4D). Previously, Wienholds et al. identified 115 conserved vertebrate miRNAs in zebrafish and carried out microarray and in situ experiment to explore temporal and spatial expression of these miRNAs [31]. Most conserved and high abundance of vertebrate miRNAs were also detected by sequencing and some miRNAs in clusters 2, 3, 4, and 5 were supported by the evidence of previously in situ hybridization experiment [31] with an expression or center nervous system preference pattern, such as miR-10a/b, miR-222a-3p, miR-9-5p/3p, and let-7a (Fig. 4A). The miRNA cluster is that multiple miRNAs are localized as clusters in the genome, transcribed in the same orientation, conserved, and often highly co-expressed [32, 33]. We also observed four miRNA clusters (miR-183/96/182, miR-18c-363, miR-17–92, and miR-430a/c/b) presenting a similar expression pattern and clustered together, and most members from miR-17–92 and miR-18c-363 clusters showed expression in brain (Fig. 4A and 4C). miR-183/96/182 cluster has been confirmed in differentiating in human retinal organoids by targeting PAX6 expression [34]. miR-430 cluster composed of multiple copies of miR-430a, c, b triplet which is conserved in fish, including zebrafish, belongs to a superfamily which also includes vertebrate miR-17–20 cluster (mir-17–92 here), and involves in brain development as well as zygotic genome activation [35, 36]. miR-430 showed a dramatical decrease in expression along with embryonic development with a large fold change in comparisons of 48 h vs. 24 h and 96 h vs. 48 h (Fig. 4B). Brain-preference expression of miRNAs, such as miR-9, showed a significant increase and peaked at 48 h compared to 24 h and 96 h (Fig. 4B). Combined evidence of brain-preference miRNAs detected in this study could provide candidate miRNAs for the following miRNA-mRNA regulatory network in nervous system development.

### miRNA-mRNA Interacting Pairs Related with Nervous System Development

**Fig. 5:**
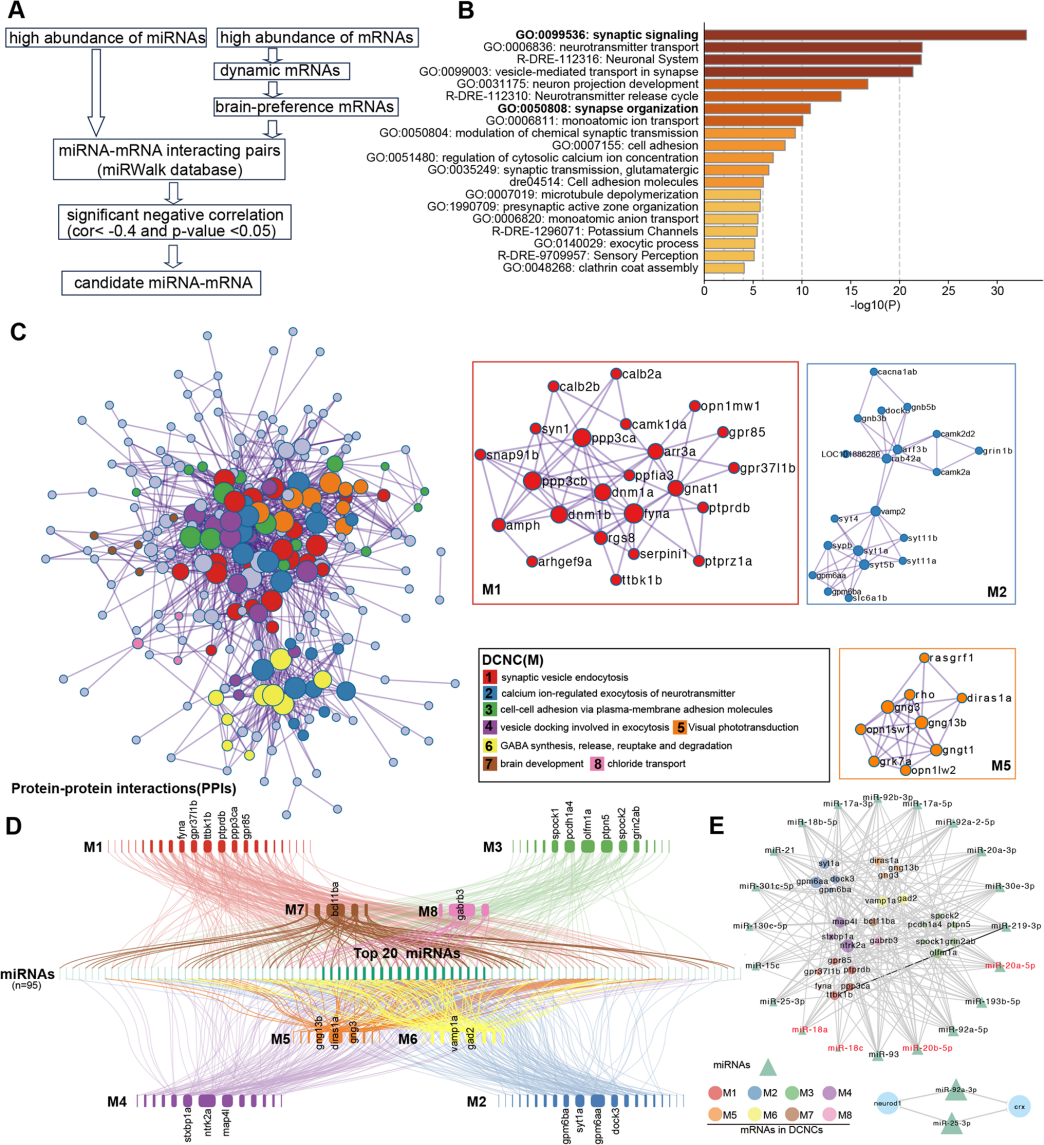
Potential miRNA-mRNA interacted pairs involved in nervous system development. **A** The workflow of screening miRNA-mRNA interacted pairs. **B** Functional enrichment analysis of predicted miRNA targets. **C** Protein–protein interaction network and densely connected network components (DCNCs). **D** Potential miRNA regulated genes in DCNCs (M1–M8) visualized with a “synteny”-like plot. Link colors and rectangle colors in up- or down-panel depict DCNC colors in panel C. Highly connected genes in each DCNC were labeled. **E** Visualization of miRNA-mRNA pairs of the top 20 connected miRNAs and highly connected genes in each DCNC and common miRNAs in regulating two key retinal differential transcription factors, *crx* and *neurod1*

We therewith integrated miRNA and mRNA expression profiles as well as the information of miRNA targets (Fig. 5A). First, we discarded those miRNAs and mRNAs with a low abundance. Dynamic expressions of mRNAs at these three time points (that is, differentially expressed genes by pairwise comparisons) and with a high preference in adult brain tissue (tau index ≥ 0.7) were selected as target genes of interest. Then we retrieved miRNA targets from the miRWalk database [20]. miRNA-mRNA pairs with a significantly negative correlation (*r* <  − 0.4 and *p* value < 0.05) in expression-level between miRNA and mRNA were selected as candidates. Finally, 131 miRNAs and 414 mRNAs were kept and resulted in 3710 miRNA-mRNA pairs (Table S5). GO enrichment analysis of these mRNAs showed that genes related with “synaptic signaling” (GO: 0099536) were most over-represented (Fig. 5B). Further protein–protein interaction (PPI) network analysis of these genes showed that most genes formed highly connected physical interactions (Fig. 5C). Then, we used the molecular complex detection (MCODE) algorithm implemented in the online tool, Metascape (https://metascape.org), to identify densely connected network components (DCNC) in this PPI network [21, 37]. This analysis resulted in eight DCNCs (designed as M1–M8, Fig. 5C). Among these DCNCs, genes in M1 were most related with “synaptic vesicle endocytosis” (e.g., *amph*, *dnm1a*/*b*, and *syn1*), and genes in M2 were most related with “calcium ion-regulated exocytosis of neurotransmitter” (e.g., *vamp2*, *camk2d2*, and *grin1b*), and genes in M5 were most related with “visual phototransduction” (e.g., *opn1lw1*, *opn1lw2*, and *rho*, Fig. 5C). We then focused on potential miRNAs that may regulate genes in these eight DCNCs and presented as the “synteny”-like plot (Fig. 5D). We observed several genes in each DCNC (labeled in Fig. 5D) were regulated by multiple miRNAs, for example, *ttbk1b* (tau tubulin kinase 1b) may be regulated by 28 miRNAs (Fig. 5D). *Ttbk1b* (orthologous to human TTBK1), a neuron-specific tubulin kinase 1b, involves in pathological phosphorylation of tau and TDP-43 and drives neurodegeneration (e.g., Alzheimer’s disease and other tauopathies) [38]. miRNAs regulating TTBK1 have been reported as miR-219-5p [39]. We also observed miR-219-3p that may target *ttbk1b* (Fig. 5E). Interacted pairs of the top 20 highly connected miRNAs and highly connected mRNAs in each DCNC were extracted and shown in Fig. 5E. Of these top 20 miRNAs, miR-18a and miR-20a belong to the miR-17–92 cluster, and miR-18c and miR-20b belong to the miR-18–363 cluster. miR-18a has been reported with a role in photoreceptor differentiation in the retina of zebrafish by regulating NeuroD [40]. *Crx* and *neurod1* are the key two transcription factors in regulating retinal differential. We found miR-25-3p and miR-92a-3p may regulate both *crx* and *neurod1* (Fig. 5E), which required further functional experiments to confirm and mechanically explore. Overall, we investigated differentially expressed miRNAs with the potential to regulate dynamic brain-preference genes identified in this study. We also highlighted densely connected network components that may warrant further investigation during brain development.

## Discussion

For the research community, we profiled both protein-coding genes and miRNAs in developing head of zebrafish at three critical windows. Our study employed rRNA-depleted enriched strategy that allowed to explore both protein-coding and non-coding RNAs, such as long non-coding RNAs and circular RNAs. Here, we aimed to systematically explore head enriched protein-coding RNAs and miRNAs and their potential regulatory networks in nervous system development, which may contribute to the understanding of neurodevelopmental system and neurodevelopmental disorder.

In comparison to the transcriptome of the whole embryo at the same time points, our data exhibited higher sensitivity in exploring dynamic brain-preference genes, as indicated by the elevated percentage of DEGs and significant enrichment in brain-preference and synapse-related gene sets. It should be also noted a situation that certain genes may show increased expression in both the whole embryo and head datasets. However, those with a larger fold change in the head, reaching the given threshold, while not meeting the threshold in the whole embryo, are categorized as head-preference genes. Therefore, our comparison and inference were based on the DEGs with a large fold change. By tissue-specific analysis, we noted that the smallest number of genes was exclusively expressed at 48 h, while the largest number of genes was exclusively expressed at 96 h and the latter were mostly involved in phototransduction (e.g., *rho*, *opn1mw1*, and *gnat1*). The rod and cone photoreceptors are sensory neurons with specialized visual pigments that initiate visual function [41]. Previous studies have shown that visual opsin proteins (rods and cones) are initially expressed around at 50 h [42], which is consistent with our observation (after 48 h).

By clustering analysis of differential expression genes identified by pairwise comparison, we detected a high expression of cell cycle-associated genes at early developmental stage. Cell cycle is the key to drive organ morphogenesis [43]. We also found a high ratio of transcription factors (TFs) including essentially TFs that control retinal differentiation and retinal synaptogenesis, such as *sox11b*, *atoh7*, *crx*, and *foxn4* [41, 44], are relatively highly expressed at 48 h. By 96 h, we found a large number of synaptic signaling-associated genes were highly expressed, such as *gabrb4* and *syn2a*. Syn2a, which belongs to the synaptic proteins, is strongly involved in neuronal development and neurotransmitter release [45]. *Mytl1a*, a homolog of human MYT1L, is a known transcription factor for brain development [46] which highly expressed at 96 h. MYT1L mutation could cause neuronal developmental disorders, including intellectual disability, autism, and attention-deficit hyperactivity disorder (ADHD) [46]. We also observed genes related with cerebellum development, such as *aldocb* (aldolase C, fructose-bisphosphate, b), a homolog of human ALDOC, are markers for Purkinje cell subpopulation [47] and showed a high expression at 96 h. *Aldoc* involves in fructose 1,6-bisphosphate metabolic process and glycolytic process. We also noted that another metabolic enzyme, *Pgk1*, which regulates glycolytic metabolism, also showed a relatively high expression at 96 h (Fig. 1E). *Pgk1* has been reported deficient in Parkinsonism patients and may contribute to nigrostriatal damage [48, 49]. Another gene, *dpysl5b* (DPYSL5 in human), a member of the collapsing response mediator protein family, is involved in neural development, and missense mutation in human could cause neurodevelopmental disorder [50].

Although we have screened numerous brain-preference genes, and most of their human orthologs have been annotated with human brain diseases, some of them still lack sufficient research. For example, *FAXC* (failed axon connections homolog, metaxin like GST domain containing; *faxca* and *faxcb* in zebrafish) has been reported in a de novo 6q16.1 deletion region identified in a 3-year-old female case by exome sequencing which may contribute to her developmental delay [51], but its expression and function were almost unknown. With the advantage of well-established genetic manipulation (e.g., CRISPR-Cas9) and almost transparent embryos in zebrafish, we also explored potential function of *snap25b* in zebrafish development using the CRISPR-Cas9 technology. *Snap25b*, an orthologous human SNAP25, is a component of SNARE complex and involves in neurotransmitter release [29]. We found that *snap25b* mutants caused little effect on larvae viability but large effect on embryonic development including increase of malformation and locomotor dysfunction. In mice, *Snap25* deficiency caused embryonic lethality while mice with *Snap25* haploinsufficiency could survive into adulthood and exhibited marked hypoactivity [52], which was consistent with our observation in zebrafish. The deletion of *Snap25* in mice could cause a strong reduction of neuron survival and impaired arborization of surviving cells [53]. Therefore, the abnormal embryos caused by *snap25b* mutation may be related with the neuronal loss.

Most miRNAs are functionally conserved in vertebrates. We identified dynamic miRNAs during head development and integrated with other development and/or tissue-related miRNA datasets. We found several miRNA clusters showed co-expression during the head development including well known for retinal development, mir-17–92. High abundance of miRNAs in brain including validated miRNAs, miR-137-3p, miR-125b-5p, miR-128-3p, miR-125a, let-7a, and miR-124-3p were identified and most have been confirmed in neural development, such as let-7 and miR-125 in fly could regulate neuronal integrity [54] and brain-enriched miR-128 regulates neurogenesis and synaptogenesis in mice [55]. Focusing on those brain-preference genes and their potential miRNAs, we identified several densely connected network components which may be critical in the head development and functional enriched in synaptic vesicle endocytosis. Among these genes, we noted *ttbk1b*, an ortholog of human TTBK1 and a neuron-specific tubulin kinase, and it could regulate neurodegeneration pathogenic genes, MAPT (encoding Tau protein) and TARDBP (encoding TDP-43 protein), via phosphorylation [38]. Regulation between miR-219 and TTBK1 in human cell line SH-SY5Y has been reported [39], suggesting other regulation predicted here may be worth further investigation.

Finally, a limitation of this study lies in the absence of experimental validation and functional exploration using genetic manipulation (e.g., CRISPR-Cas9 and new transgenic lines) for both more brain-preference genes and miRNA-mRNA interactions.

## Conclusion

In summary, this study provides a comprehensive transcriptome resource for zebrafish investigators and also offers novel insights into dynamic mRNA and miRNAs as well as miRNA-mRNA networks during the head development, which will benefit for conducting further human disease modeling.

## Supplementary Information

Below is the link to the electronic supplementary material.

Supplementary file1 (DOCX 1747 KB)

Supplementary file2 (XLSX 530 KB)

Supplementary file3 (XLSX 102 KB)

Supplementary file4 (XLSX 12 KB)

Supplementary file5 (XLSX 19 KB)

Supplementary file6 (XLSX 1003 KB)

Supplementary file7 (XLSX 10 KB)

## Data Availability

CNGBdb; https://db.cngb.org.
