# Supplementary material for: Integrative mRNA and miRNA Expression Profiles from Developing Zebrafish Head Highlight Brain-Preference Genes and Regulatory Networks
Source: Mol Neurobiol. 2024 Jul 31;62(2):2148–62. doi: 10.1007/s12035-024-04364-5 (PMC11772381; doi:10.1007/s12035-024-04364-5)
Supplement: Supplementary file 1 — Supplementary file1 (DOCX 1747 KB) [file 12035_2024_4364_MOESM1_ESM.docx]

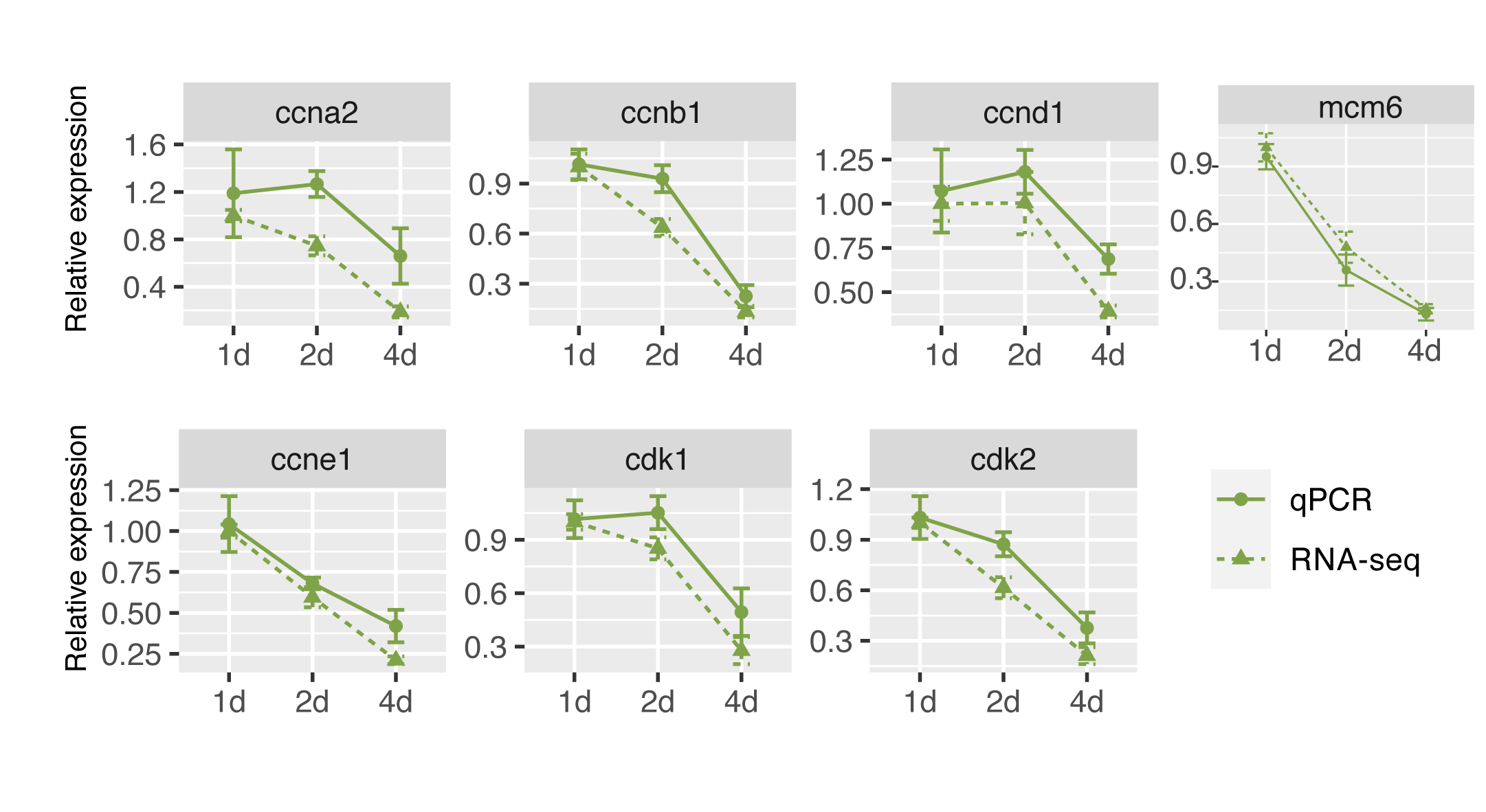


**Figure S1. RT-qPCR validation of seven cell-cycle related genes during the head development in zebrafish**.


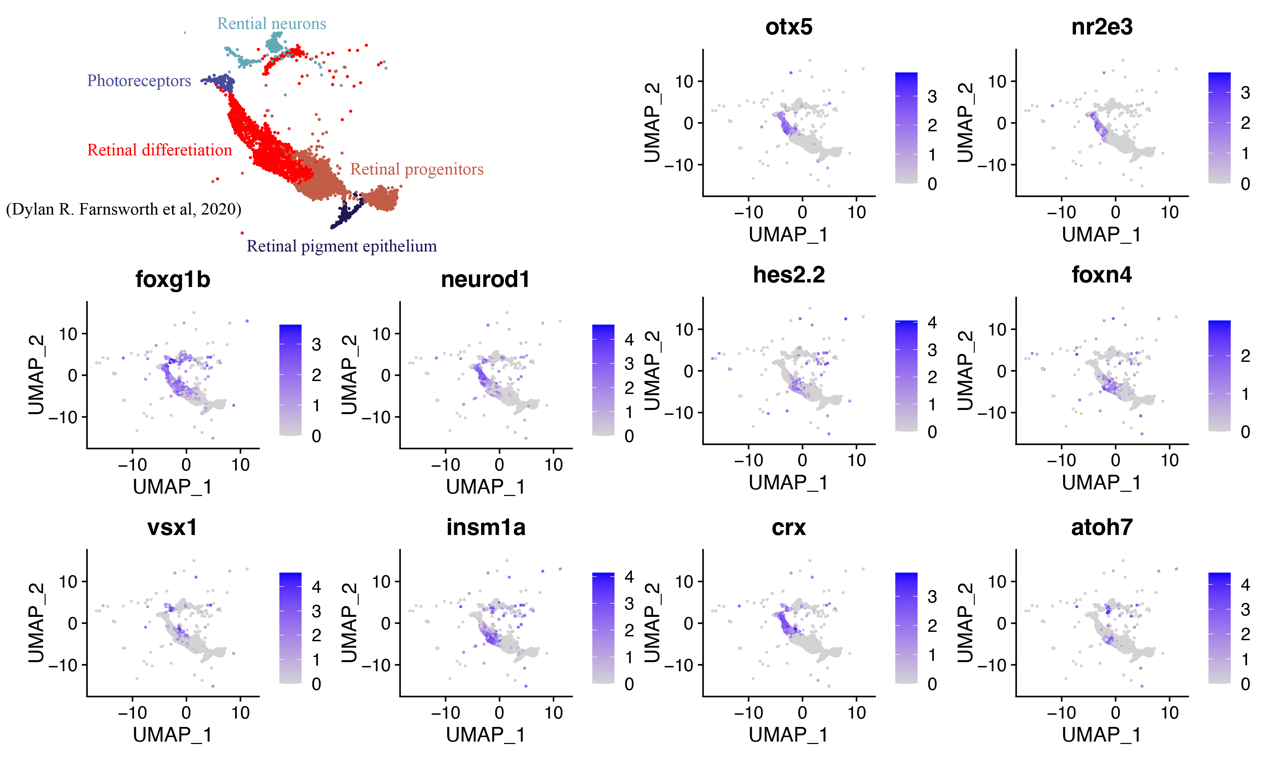


**Figure S2. Expression of 10 TFs in cluster 4 from Fig. 1c based on single-cell RNA sequencing of developmental zebrafish**. Only cell clusters related with eye development were shown. UMAP plot of these cell clusters was retrieved from the study.


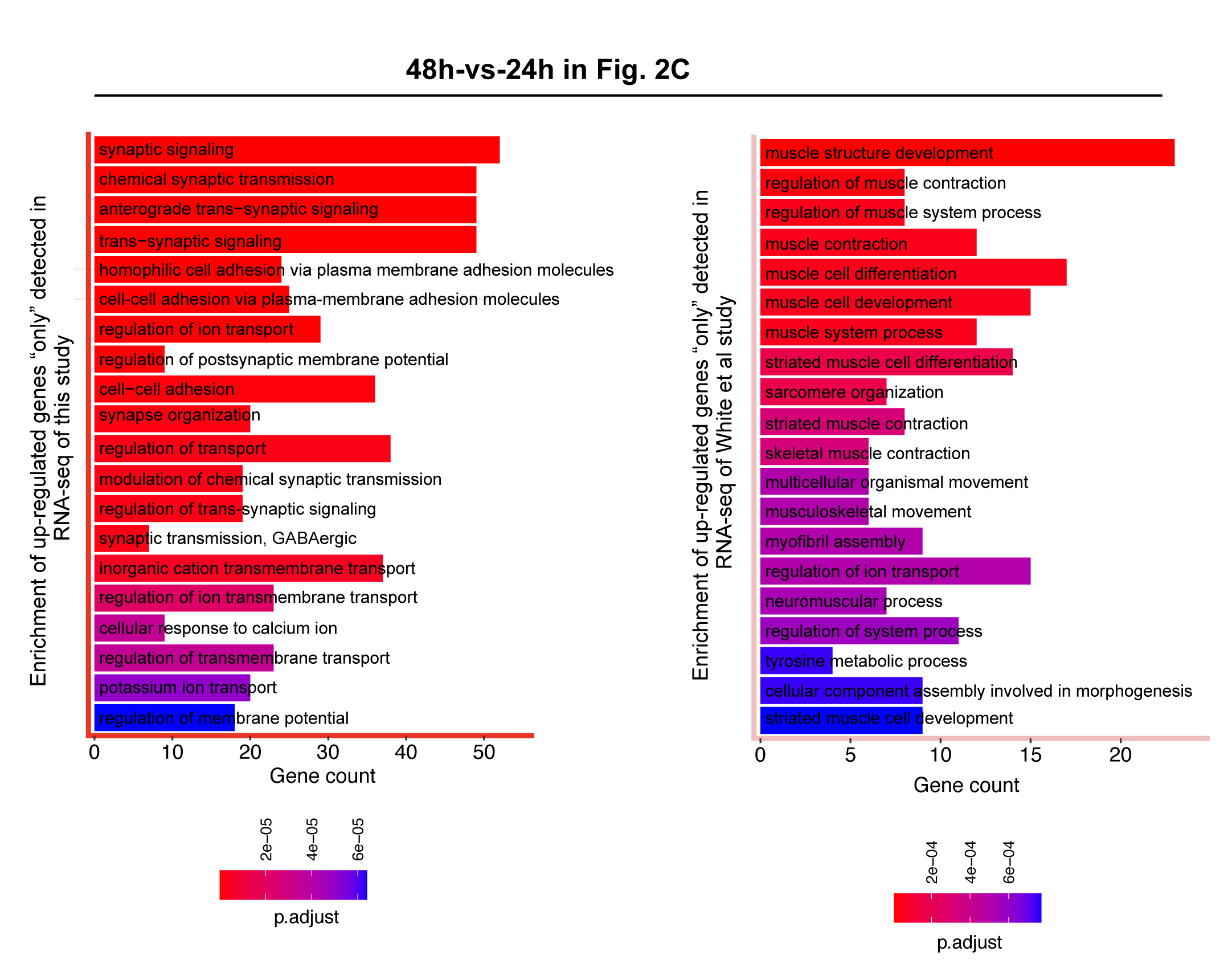


**Figure S3. GO enrichment (Biological Process) of genes in Fig. 2C of the main text by comparison between 48 h and 24 h**. Left panel is enrichment of up-regulated genes only detected in head transcriptome the given criterion. Right panel is enrichment of up-regulated genes only detected in White et al study at the given criterion.


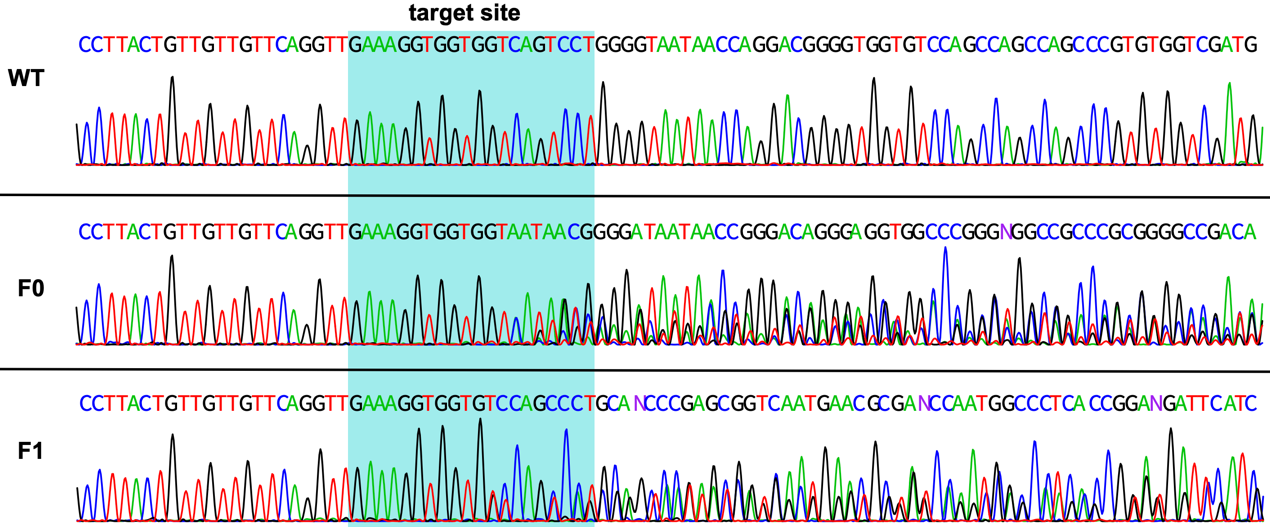


**Figure S4. Sanger sequencing confirm the *snap25b* mutations by CRISPR-Cas9 technology in F0 and F1 zebrafish**. Green box shade in the wild-type (WT) was target site.
